# 

**Published:** 1962-04

**Authors:** 


### MEDICAL JOURNAL OF THE SOUTH-WEST

*(Incorporating the Bristol Medico-Chirurgical Journal)*

Established 1883

VOL. 77 (ii) April 1962 NO. 284

### *Contents*

IATROGENIC LIVER DISEASE . . . . . *A. E. Read* 41

CONGENITAL MALFORMATIONS . . *Beryl D. Corner* 46

CONGENITAL MALFORMATIONS . . . *N. J. Brown* 53

UNUSUAL TYPE OF BACTERIAL ENDOCARDITIS IN A CASE OF MYOCARDIAL INFARCTION . . *Clinico–Pathological Conference* 60

NOTES AND NEWS . . . . . . . . . 65

EXAMINATION RESULTS . . . . . . . . 65

APPOINTMENTS . . . . . . . . . 65

NEWS FROM THE SOCIETIES . . . . . . . 67

*PUBLISHED QUARTERLY*

*ANNUAL SUBSCRIPTION* 16/- *POST FREE*

PUBLISHED UNDER THE AUSPICES OF THE
BRISTOL MEDICO-CHIRURGICAL SOCIETY

### *Editorial Committee*

Dr. N. J. Brown, Southmead Hospital, Bristol. *Joint Editor.*

Dr. A. B. Raper, Department of Pathology, Bristol Royal Infirmary. *Joint Editor.*

Dr. J. Macrae, Ham Green Hospital.

Dr. R. C. Wofinden, Central Clinic, Bristol.

Mr. A. E. S. Roberts, Medical Library, University of Bristol. *Secretary.*

### *Local Correspondents*

*Bath* . . Mr. A. D. Bateman, 3 The Circus, Bath.

*Exeter* . . Dr. L. N. Jackson, Mount Jocelyn, Crediton, Devon.

*Gloucester* . Dr. R. F. Jarrett, 9 College Green, Gloucester.

*Plymouth* . Dr. C. R. Croft, Lexden, Hartley Av., Plymouth.

*Taunton* . Dr. M. McAlley, 1 The Crescent, Taunton.

*Torquay* . Dr. A. Robinson Thomas, 20 Devon Square, Newton Abbot, Devon.

*Truro* . . Dr. F. D. M. Hocking, St. Andrews, Perranporth, Cornwall.

*Yeovil* . . Mr. J. A. Vere Nicoll, Knapp House, Yeovil, Somerset.

### NOTICE TO CONTRIBUTORS

Original articles are invited, provided they have not been published elsewhere. Notes of forthcoming events, meetings held, degrees conferred, staff appointments, and other local news are welcomed. The editorial committee reserves the right to make literary corrections.

Illustrations should always be supplied ready for reproduction. All re-touchings or other operations required will be charged to the author. Line drawings should be drawn in Indian ink. We regret that we must ask authors to pay for making blocks, if this is necessary.

J. W. ARROWSMITH LTD., WINTERSTOKE ROAD,
BRISTOL.

### *Notes and News*

NEW YEAR HONOURS

*O.B.E.:* Dr. G. D. Kersley, T.D., M.D., F.R.C.P. Consultant Physician, Royal United Hospitals, Bath.

### *Examination Results*

UNIVERSITY OF BRISTOL

*Ph.D.* (Faculty of Medicine): W. F. Butler, W. A. G. Charleston.
*D.P.H.:* L. Ziaee.
*M.B., Ch.B.:* Audrey K. Ball, P. J. Froud, R. A. Jones, H. V. Poku, D. A. O. Sutton, P. J. Thomas.

### *Appointments*

Surgeon Captain H. L. Cleave, O.B.E., R.N., has been appointed an Honorary Surgeon to the Queen.

D. N. Walder, M.D., CH.B. (Bristol), F.R.C.S., F.R.C.S.E., has been appointed Reader in experimental surgery, University of Durham.

W. B. Spry, M.B., CH.B. (Bristol), D.P.M., has been appointed Consultant Psychiatrist, Herefordshire Hospital Group.

### *UNITED BRISTOL HOSPITALS*

D. W. Barritt, M.D., M.R.C.P., Consultant Physician.
D. R. Coles, M.D., M.R.C.P., Consultant Physician.
R. F. N. Duke, B.M., B.CH., F.R.C.S., Senior Registrar in Orthopaedics.
J. P. Davie, M.B., CH.B., D.A., Registrar in Anaesthetics.
S. V. Mahajani, B.D.S., Registrar in Dental Surgery.
G. H. Bevan, M.B., B.S., Registrar in Ophthalmology.
A. N. Henry, M.B., B.CH., F.R.C.S.I., F.R.C.S., Registrar in Orthopaedics.
C. F. McCarthy, M.D., B.CH., M.R.C.P.I., M.R.C.P., Registrar in Medicine.
D. R. Langley, B.SC., M.B., B.CH., Registrar in Anaesthetics.
Miss M. M. Walshe, M.B., B.S., Registrar in Dermatology.
P. Keane, M.B., CH.B., Registrar in Pathology.
G. Jacob, M.B., B.S., M.R.C.P.E., Registrar in Cardiology.
J. Moule, M.B., CH.B., Registrar in Radiology.
J. Smith, M.B., CH.B., Registrar in Radiology.
D. B. Evans, M.B., B.CH., Registrar in Medicine.

### *SOUTH WESTERN REGIONAL HOSPITAL BOARD*

*November* 1961 *to January* 1962

### *BRISTOL*

| *Name* | *Appointment* | *Formerly* |
|---|---|---|
| JANCAR, J., M.D., D.P.M. | Consultant Psychiatrist, Stoke Park and Hortham/Brentry Hospital Groups. | Assistant Psychiatrist, Stoke Park Hospital Group. |
| DRURY, P. M. E., M.B., B.S. (LOND.) | Anaesthetic Registrar, Southmead Hospital. | Clinical Officer in Anaesthetics, Catterick Military Hospital, Yorks. |

### *NORTH GLOUCESTERSHIRE*

| *Name* | *Appointment* | *Formerly* |
|---|---|---|
| BLATCHLEY, W. R., M.B., B.S. (LOND.) | Clinical Assistant to the Rheumatism Clinic in Gloucester for 1 weekly session (re-appointed) | Is in General Practice in Gloucester. |

### *BATH*

| | | |
|---|---|---|
| WEBSTER, F. L., M.B., Ch.B. (BRISTOL)<br>WILSON, EDITH. J., M.B., Ch.B. (ST. ANDREWS), D.A. | Anaesthetic Registrars to the Bath Group of Hospitals. | Locum Anaesthetic Registrar, Bath Group of Hospitals.<br>Anaesthetic Registrar, Portsmouth Group of Hospitals. |
| KRISHNAMURTHY, B. P., M.B., B.S. (MYSORE), L.M.S.S..A. (LOND.) | Orthopaedic Registrar, Bath Group of Hospitals. | Locum Orthopaedic Registrar, North Staffs Royal Infirmary. |

### *DEVON AND EXETER*

| | | |
|---|---|---|
| LUPPRIAN, K., M.B., B.S., D.A., F.F.A.R.C.S. | Consultant Anaesthetist, Devon and Exeter Clinical Area. | Senior Anaesthetic Registrar, St. Thomas's Hospital, London. |
| VOWLES, K. D. J., M.B., Ch.B., F.R.C.S. | Consultant Surgeon, Devon and Exeter Clinical Area | Lecturer in Surgery, University of Bristol and Honorary Senior Registrar, United Bristol Hospitals and this Board. |
| ASHTON, F., M.A., M.D., | Assistant Physician to the Geriatric Service in the Devon and Exeter Clinical Area. | Registrar in Geriatrics, Southampton Group of Hospitals. |
| SEATON, D. N., M.B., B.CHIR., (CANTAB). | Clinical Assistant in General Medicine, Tiverton and District Hospital for 3 weekly Sessions. | Is in General Practice in Tiverton. |
| SHACKLETON, JEAN, M.B., CH.B. (MANCHESTER) D.C.H., D.OBST.R.C.O.G. | Paediatric Registrar, Royal Devon and Exeter Hospital/The Westminster Hospital, London (joint appointment with The Westminster Hospital). | Locum Paediatric Registrar, Westminster Children's Hospital, London. |

### *PLYMOUTH*

| | | |
|---|---|---|
| LEATHER, H. M., M.D., M.R.C.P. | Consultant in General Medicine, Plymouth Clinical Area. | Senior Medical Registrar, Medical Professorial Unit, Bristol Royal Infirmary. |
| MUKHOPADHYAY, S. D., M.B., B.S. (CALCUTTA) F.R.C.S. (EDIN.) | Surgical Registrar, South Devon and East Cornwall Hospital, Plymouth. | — |
| BHONSLE, R. S., M.B., B.S. (BOMBAY). | Registrar in E.N.T. Surgery, South Devon and East Cornwall. Hospital, Plymouth. (re-appointed). | Registrar in E.N.T. Surgery, South Devon and East Cornwall Hospital, Plymouth. |

### *WEST CORNWALL*

| | | |
|---|---|---|
| RAY, P., M.B., B.S. (CALCUTTA). | Surgical Registrar, Royal Cornwall Infirmary, Truro. | Locum appointments. |

